# Prevention of UVA-Induced Oxidative Damage in Human Dermal Fibroblasts by New UV Filters, Assessed Using a Novel *In Vitro* Experimental System

**DOI:** 10.1371/journal.pone.0083401

**Published:** 2014-01-07

**Authors:** Francesca Brugè, Luca Tiano, Paola Astolfi, Monica Emanuelli, Elisabetta Damiani

**Affiliations:** 1 Dipartimento Scienze Cliniche Specialistiche ed Odontostomatologiche, Università Politecnica delle Marche, Ancona, Italy; 2 Dipartimento Scienze e Ingegneria della Materia, dell'Ambiente ed Urbanistica, Università Politecnica delle Marche, Ancona, Italy; 3 Dipartimento Scienze della Vita e dell'Ambiente, Università Politecnica delle Marche, Ancona, Italy; University of Tennessee, United States of America

## Abstract

**Background:**

UVA rays present in sunlight are able to reach the dermal skin layer generating reactive oxygen species (ROS) responsible for oxidative damage, alterations in gene expression, DNA damage, leading to cell inflammation, photo-ageing/-carcinogenesis. Sunscreens contain UV filters as active ingredients that absorb/reflect/dissipate UV radiation: their efficiency depends on their spectral profile and photostability which should then be reflected in biological protection of underlying skin.

**Methods:**

A set of new UV filters was synthesized, and the most photostable one was compared to BMDBM, a widely used UVA filter. Cultured human dermal fibroblasts were exposed to UVA radiation which was filtered by a base cream containing or not UV filters placed above cell culture wells. The endpoints measured were: cell viability (MTT assay), ROS generation (DCFH-DA assay), mitochondrial function (JC-1 assay), DNA integrity (Comet assay) and gene expression (MMP-1, COL1A1) by RT-qPCR.

**Results:**

The new UV filter resulted more efficient than BMDBM in preserving cell viability, mitochondrial functionality and oxidative DNA damage, despite similar inhibition levels of intracellular ROS. Moreover, expression of genes involved in dermal photoageing were positively affected by the filtering action of the tested molecules.

**Conclusions:**

The experimental model proposed was able to validate the efficacy of the new UV filter, taking into account important cellular events related to UV-induced intracellular oxidative stress, often underestimated in the assessments of these compounds.

**General Significance:**

The model may be used to compare the actual biological protection of commercial sunscreens and suncare products aside from their SPF and UVA-PF values.

## Introduction

UV radiation represents 5% of the total solar radiation reaching the earth's surface, and is divided into two spectral regions: UVA (320–400 nm) and UVB (290–320 nm) constituting ∼96% and ∼4%, respectively, and both are responsible for the carcinogenic effect associated with sunlight overexposure [1]. Differently from UVB, UVA rays are able to penetrate further into the dermal layers of skin where they are absorbed by skin chromophores triggering the generation of reactive oxygen species (ROS) in the resident dermal fibroblasts and in extra-cellular structures [2]. This contributes to oxidative damage, alterations in gene expression, DNA damage, ultimately leading to cell inflammation, photoageing and photocarcinogenesis [3]–[5]. It is no surprise then, that in recent times, the necessity to combine UVA protection to the already known UVB protection of sunscreens has become of major importance, leading to the development of sunscreens containing broadband UVB/A filters.

UV filters are the active ingredients of sunscreens able to absorb/reflect/dissipate UV radiation thus reducing the amount of UV light reaching the viable skin layers [6], and two important requisites which determine their efficacy are their spectral profile and their photostability. Their absorbance spectra should remain unaltered throughout the whole exposure period guaranteeing uniform and sufficient UVA/B coverage, which implies that they should be photochemically stable. They should not breakdown after UV absorption since this would lead to a loss in absorbance and consequently to reduced photoprotection of the sunscreens containing them. In addition, any photoproducts released following degradation, that may also comprise ROS, could be toxic, irritant and cause allergic reactions responsible for skin alterations. Most UV filters are sufficiently photochemically stable, however some commonly used ones are not. For example, butyl methoxydibenzoylmethane (BMDBM), the most widely used UVA filter in sunscreens worldwide and nowadays also in daily face creams, is inherently photounstable, breaking down to form free radicals following UVA exposure [7]–[9]. For this reason, it is common practice, albeit not always, to co-formulate it with photostabilizers such as octocrylene, methylbenzylidene camphor or bis-ethylhexyloxyphenol methoxyphenyl triazine, to reduce its photoinstability [10].

Following our on-going studies on the photostability assessment of sunscreens and UV filters and on our pursuit of new UV filters [11]–[19], we recently came across a class of compounds, namely benzoxazine nitrones, that absorb in the UVB and partly in the UVA region. However, nitrones are notoriously known to be photosensitive and under UV irradiation they photo-rearrange and decompose with consequent decrease in UV absorbance [20], [21]. Therefore a set of compounds with similar structure, but without the nitrone group responsible for the photoinstability observed, and with different substituents typically found in UV filters, were synthesized. Their photostability was first assessed *in vitro* in liposomes, and the most promising compound was then formulated in a cream and its efficacy was tested and compared to BMDBM. For this latter purpose, a cell culture system consisting of human dermal fibroblasts was employed and exposed to UVA light in the presence or absence of the formulations, which were spread on quartz petri-dishes placed above the cells but not in direct contact with them. In fact, a good UVA filter should not penetrate the epidermal layer and should screen out UVA rays as much as possible, in order to protect the underlying dermal fibroblasts from UVA damage. Several end-points which directly reflect UVA-induced oxidative damage were determined, such as intracellular ROS production, mitochondrial function, DNA integrity. The mRNA expression levels of two important genes linked to photoageing were also investigated: MMP-1, the major enzyme responsible for collagen degradation in the dermis [22], and COL1A1, the gene encoding for synthesis of α1(I)-procollagen, a component of type-I collagen [23]. Using the simple *in vitro* experimental method proposed, the efficacy of UV filters and sunscreens at the cellular and molecular level, under conditions which closely mimic their actual application and exposure, can be investigated.

## Materials and Methods

Carboxy-2′,7′-dichlorofluorescein diacetate (carboxy-DCFH-DA), 5,5′,6,6′-tetrachloro-1,1′,3,3′ tetraethylbenzimidazolcarbocyanine iodide (JC-1), and propidium iodide were purchased from Molecular Probes (Invitrogen). Formamido-pyrimidine-glycosylase (FPG) was kindly donated by Dr. A. Collins. Primary cultures of human dermal fibroblasts (HDF) were purchased from the Istituto Zooprofilattico Sperimentale, Brescia, Italy. Minimum Essential Medium (MEM) for cell cultures was obtained from GIBCO (Invitrogen), whereas all other cell culture reagents were from PAN biotech, GmbH. 2-Aminophenol, 2-amino-4-*tert*-butylphenol, 2-amino-4-methoxyphenol, 2-bromoacetophenone, 2-bromo-4′-acetophenone, 1,3-diacetylbenzene as well as all other reagents were purchased from Sigma-Aldrich Chemical Co. (Milan, Italy). 1,4-Bis(bromoacetyl)benzene was obtained from AlCl_3_-catalyzed bromination of 1,3-diacetylbenzene as reported in [24]. Solvents were purchased from Carlo Erba and were used without further purification.

### Synthesis of Benzoxazines 1–5: General Procedure

**Figure 1:**
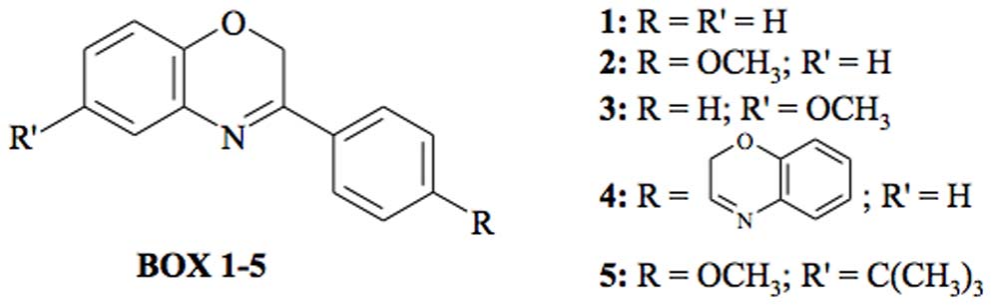
Chemical structures of compounds studied.

Benzoxazines 1–5 (abbreviated to BOX 1–5, Fig. 1) were synthesized following a procedure described in the literature [25]: to a solution of the appropriate aminophenol (1 mmol, except for BOX 4 where 2 mmol were used) in dichloromethane (6 ml), aqueous potassium carbonate solution (4 ml, 20%) and tetrabutylammonium hydrogensulfate (0.05 mmol) were added. The resulting two-phase mixture was magnetically stirred and to this emulsion a solution of the appropriate acetophenone (1 mmol) in dichloromethane (2 ml) was added dropwise. The mixture was stirred for 1 h at room temperature. The reaction course was checked by thin layer chromatography (TLC) using cyclohexane/ethyl acetate 7/3 as the eluant. The organic layer was separated and washed with water (3×25 ml), dried over sodium sulphate and evaporated to dryness. The crude residue was purified by column chromatography using cyclohexane/ethyl acetate 7/3.

^1^H NMR spectra were recorded in CDCl_3_ on a Varian 400 spectrometer. J values are given in Hertz and residual protic solvent CHCl_3_ (δ_H_ = 7.26 ppm) was used as the internal reference. ^13^C NMR spectra were recorded in CDCl_3_ at 100 MHz, on a Varian 400 spectrometer using the central resonance of CDCl_3_ (δ_C_ = 77.16 ppm) as the internal reference. IR spectra were recorded in the solid state on a Perkin-Elmer MGX1 spectrophotometer equipped with Spectra Tech. Mass spectra were recorded on a Carlo Erba QMD 1000 mass spectrometer in EI+ mode.

### Liposome preparation

Stock solutions of BOX 1–5 (5 mM) were each prepared in chloroform and 0.0075 ml were added to a glass test-tube kept on ice and mixed with 0.04 ml L-α-phosphatidylcholine (P2772: Type XI-E). The solvent was thoroughly evaporated under a stream of nitrogen and the resulting lipid film was dispersed in 1.5 ml PBS/EDTA (5 mM phosphate buffer, 0.9% NaCl, 0.1 mM EDTA, pH 7.4) and vortexed for 10 min until a white, homogeneous, opalescent suspension was obtained.

### Cell cultures

HDF were cultured in 25 cm^2^ flasks in MEM supplemented with 10% SERA PLUS special processed fetal bovine serum, penicillin (100 U/ml), streptomycin (100 µg/ml) and L-glutamine (2 mM), at 37°C in a 5% CO_2_ Heraeus BB15 incubator (Thermo Scientific) and in humidified atmosphere. For cell culture maintenance, medium was changed every 2–3 days and cells were passaged at 80% confluence by trypsinization. For the experiments, cells were used between 8^th^–12^th^ passage and were seeded at an optimal density of 14×10^3^ cell/cm^2^.

### Formulation preparation

The two UV-filters, BOX 2 and BMDBM were formulated separately in the same oil-in-water formulation obtained as briefly described: phase A (14% PEG-8 beeswax, 19% caprylic/capric triglyceride, 7% olive oil, 6% UV filter, 1% tocopherol acetate, 0.4% sodium hydroxide) and phase B (0.5% EDTA, 0.2% hydroxyethyl cellulose and deionized water making up 100%) were heated separately to 65°C under continuous stirring. B was then added to A under continuous, vigorous stirring at 65°C for 2 min, followed by cooling at room temperature under continuous stirring until a homogenous consistency was reached. The control cream was prepared similarly but without addition of UV filters.

### UV exposure procedures

As UVA irradiating source, a Philips Original Home Solarium sun lamp (model HB 406/A; Philips, Groningen, Holland) equipped with a 400 W ozone-free Philips HPA lamp, UV type 3, delivering a flux of 23 mW/cm^2^ between 300 and 400 nm, at a distance of 20 cm from the samples, was used. The dose of UVA received from above by the samples was measured with a UV Power Pack Radiometer (EIT Inc, Sterling, USA), while the emission spectrum was checked using a StellarNet portable spectroradiometer (Tampa, FL, USA) and is reported elsewhere [19].

For exposure of liposomes, 0.7 ml from each liposomal suspension was kept in the dark while another 0.7 ml was transferred into a 24 multi-well plate for cell cultures, covered with a 2 mm thick quartz slab and placed on a brass block embedded on ice and irradiated for 15 min (275 kJ/m^2^). This UVA dose is approximately equivalent to about 90 min of sunshine at the French Riviera (Nice) in summer at noon [26].

For irradiation of formulations, the protocol described in [13] was used. Briefly, an amount equivalent to 50±2 mg (checked by weighing), as recommended by the COLIPA sun protection factor test method [27], was spread onto 25 cm^2^ glass plates with a gloved finger, left to dry for 30 min in the dark, and then the plates were exposed to 15 min UVA as described above. The unexposed samples were kept in the dark for the same amount of time as the exposed ones.

**Figure 2:**
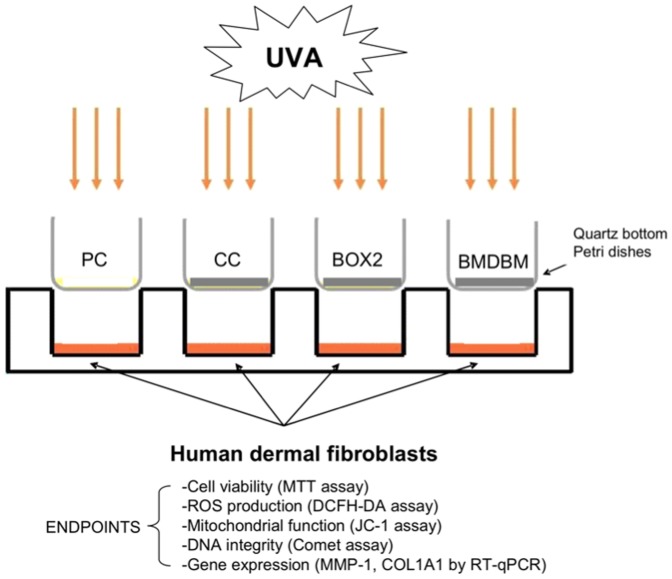
Experimental design. Formulations (BOX2, BMDBM) or control cream (CC) (2 mg/cm^2^) were spread on quartz bottom petri dishes and placed on top of the wells of cell-culture plates containing human dermal fibroblasts. The positive control (PC) was left uncoated. Cells were irradiated from above with UVA for varying lengths of time according to each assay protocol.

Cells grown on a 6-well culture dish for UVA irradiation, were first washed with phosphate buffered saline (PBS) and covered with a thin layer of PBS prior to exposure. The formulations (2 mg/cm^2^) were spread onto quartz-bottom petri dishes (designed for us by Highborn Technology, China) of exactly the same dimensions as the cell culture plates and placed on top of the wells prior to irradiation. The cells were then either not exposed (negative control), or exposed to the UVA source as described above for varying lengths of time according to each assay protocol, through an uncoated quartz-bottom petri dish (positive control, PC) or through ones coated with formulations or control cream (CC), as in the experimental design summarized in Fig. 2.

### Optical absorption spectra

After irradiation of liposomal suspensions, 0.6 ml were collected from each well and vortexed thoroughly for 2 min with the same volume of ethyl acetate for extraction of the UV-filters. The same procedure was also carried out on the non-irradiated samples. The top organic layer was then separated after centrifugation and its absorption spectrum was measured on a Shimadzu UV-2401PC spectrophotometer against a blank containing ethyl acetate.

After irradiation of formulations, the glass plates were placed in beakers and immersed in 10 ml ethyl acetate for 30 min with manual shaking every 10 min for maximum extraction of the UV-filters. From the organic solution, 0.05 ml were added to 2.45 ml ethyl acetate in a quartz cuvette and its absorption spectra was measured against a blank containing ethyl acetate on the same spectrophotometer as above.

### Cell viability assay

For this assay, 96-well cell culture plates were used and the quartz petri dishes with or without formulations were placed on the four corners and on the middle of the plate, such that each covered 9 wells, and irradiated as reported above. After exposure to UVA for 20 min (364 kJ/m^2^), PBS was removed and replaced with culture medium and cells were either analysed immediately or incubated for 24 h prior to analysis. Cell viability was measured using the MTT assay: the tetrazolium salt MTT, is reduced by intracellular dehydrogenases of viable cells leading to the formation of purple formazan crystals. For the assay, cells were washed with PBS and incubated for 2 h at 37°C with MTT salt solution (0.5 mg/ml) in RPMI medium without phenol red and with 10% FBS. The MTT solution was then replaced with DMSO prior to reading the optical density at 550 nm on a microplate reader (Synergy HT, Biotek, Winooski, VT, USA) [28]. Cell viability was expressed as a percentage of live cells compared to the unexposed control.

### Intracellular ROS assay

This assay uses carboxy-DCFH-DA, a non-polar ROS-index probe which readily diffuses across cell membranes where it is hydrolyzed by intracellular esterases to the non-fluorescent polar derivative, carboxy-DCFH. In the presence of ROS, carboxy-DCFH is oxidized to carboxy-DCF which is highly fluorescent and whose emission maximum can be monitored at 520 nm [29].

After UVA exposure for 20 min, PBS was removed, cells were detached by trypsinization, MEM was re-added, and cell suspension was centrifuged at 800 *g/*0 min. After removal of the supernatant, the cell pellet was re-suspended in 0.2 ml carboxy-DCFH-DA (final concentration 10 µM in PBS) and incubated in the dark for 15 min at 37°C. PBS (1 ml) was then added followed by centrifugation at 800 *g/*10 min. The supernatant was discarded and the cell pellet was re-suspended in 0.5 ml PBS and kept on ice in the dark. The samples were transferred to cytometry tubes and 10 µg/ml of propidium iodide were added prior to flow cytometry readings. Fluorescence of the labelled cells was measured on a Coulter EPICS XL flow cytometer (Coulter) using an excitation wavelength of 488 nm. Emissions were recorded using FL-1 for carboxy-DCF and FL-3 for propidium iodide, using photomultiplier 234 tubes (PMT) set at 600 and 750 volts, respectively. Fluorescence intensity was recorded on an average of 10,000 propidium-negative cells from each sample. Total DCF fluorescence activity was calculated using win.mdi 2.9.

### Mitochondrial membrane potential assay

Membrane potential, ΔΨ_m_ was analyzed using the Nernstian probe JC-1 which allows direct measurement of ΔΨ_m_ either in intact cells or isolated mitochondria [30]. This probe selectively enters mitochondria and in normal functioning mitochondria with a high ΔΨ_m_ it is found in its aggregate form emitting red fluorescence (590 nm) upon excitation at 488 nm. When membrane potential drops, it is found in a monomeric form in the cytoplasm emitting green fluorescence. Therefore, depending on ΔΨ_m_, JC-1 is able to form J-aggregates that cause a large shift in emission to 590 nm (red/orange), therefore the colour of the dye changes reversibly from green to orange as the mitochondrial membrane becomes more polarized (above values around 80–100 mV) [31].

After 20 min UVA exposure, the same steps as for the carboxy-DCFH-DA assay above were adopted up to the first centrifugation. Cells were then re-suspended in 0.5 ml of JC-1 (final concentration 1.9 µM in MEM) and incubated in the dark for 15 min at 37°C. PBS (0.5 ml) was then added, followed by centrifugation at 800 g/10 min. The cell pellet was re-suspended in 0.5 ml PBS in the dark, and then transferred to cytometry tubes prior to reading in the Coulter flow cytometer as reported above. Cells were analyzed using FL-1 and FL-2 emissions detected using PMT set at 572 and 580 volts respectively. Compensation was set at FL1-FL2 3.5%; FL2-FL1 20.8%. A minimum of 10,000 events were recorded. Mitochondrial depolarization was evaluated in terms of percentage of cells showing low red fluorescence, proportional to ΔΨ_m_, using win.mdi 2.9.

### Comet assay

After UVA exposure for 20 min, PBS was removed, cells were detached, MEM was added and cells were counted in a Kova Glasstic Slide 10 with grid chamber. Aliquots containing 10,000 cells from each sample were transferred to eppendorf tubes and centrifuged for 10 min at 800 *g*/4°C. The supernatant was removed and cells were resuspended in 0.7% low melting agarose from which 0.035 ml were taken and placed on pre-coated, high throughput, comet assay slides (Trevigen). The microgels on the slides were then allowed to solidify at 4°C. Subsequently, the slides were immersed overnight at 4°C in the dark, in ice-cold, freshly prepared lysis solution (2.5 M NaCl, 100 mM Na_2_EDTA, 10 mM Tris-HCl, 1% Triton X-100 and 10% DMSO, adjusted to pH 10). This was followed by washing twice with EndoBuffer (EB) for 15 min in the dark at 4°C and then the DNA repair enzyme FPG (dilution 1∶3000) for detection of oxidized purines, was added to the appropriate slides as previously described [32]. The slides for the classic version of the comet assay lacked FPG. The slides were incubated in a humidified chamber for 45 min at 37°C, followed by air-drying for 15 min at 4°C, washing in EB and equilibrating in freshly prepared alkaline buffer (1 mM Na_2_EDTA, pH 13) for 30 min. Electrophoresis was then performed for 20 min at 1 V/cm in the same buffer. After neutralization in Tris buffer (pH 7.5) and dehydration in 75% methanol, the DNA on each slide was stained with 0.015 ml ethidium bromide (20 µg/ml) and the comets were analyzed using fluorescence microscopy as previously reported [18]. For each comet, data relative to tail length (TL), tail migration (TMi), percent tail DNA (TI) and tail moment (TM) were recorded.

### Assessment of gene expression by RT-qPCR

After 24 h post-irradiation with UVA for 15 min, total RNA was extracted from the cells using SV (Spin or Vacuum) Total RNA Isolation System (Promega) and its concentration and purity were determined by UV spectrophotometry. Ribosomal RNA band integrity was evaluated by conventional denaturing agarose RNA electrophoresis [33]. Samples were then subjected to RT-qPCR as previously described [34]. Briefly, cDNA synthesis was performed using iScriptTMcDNA Synthesis Kit (Bio-Rad) according to the manufacturer's instructions followed by qPCR which was carried out with SYBR green dye technique (iQTM Supermix, Biorad) on a MyiQ Single Color Real-Time PCR Detection System (Bio-Rad). At least three biological and two technical replicates per biological replicate were performed. The primers sequences for the genes of interest, MMP-1 and COL1-A1, as well as for the reference genes, GAPDH and SDHA previously determined to be the most suitable ones for UVA studies on HDF, are those reported in [34]. The mRNA expression of MMP-1 and COL1A1 in UVA treated cells were calculated relative to the expression of these genes in control fibroblasts (non-irradiated), according to the delta-delta C_t_ method (2^−ΔΔCt^). The results were analyzed by iQ5 Software (Bio-Rad) and the mean of the normalized expression values from at least three independent experiments were calculated for data analysis.

### Statistical analysis

Statistical analysis was performed using the Student's t-test for all assays which showed parametric distribution of data, except for gene expression analysis where the Mann-Whitney U test was used. This latter test was used because the Shapiro-Wilk test showed non-parametric distribution of the gene analysis data. Data analysis was performed using the R Version 3.0.2 (Free Programming Environment for Data Analysis and Graphics). A value of p≤0.05 (*) or of p≤0.01 (**) were considered significant.

## Results

### Characterization of BOX 1–5

All the ^1^H NMR, ^13^C NMR, IR and mass spectra of BOX 1–5 reported below, confirmed their respective chemical structures:

*3-Phenyl-1,4-benzoxazine* ***1*** *(BOX 1)*: ^1^H NMR (400, CDCl_3_, 25°C) δ = 5.08 (s, 2H), 6.92 (d, 1H, *J* = 7.8 Hz), 7.44 (d, 1H, *J* = 7.8 Hz), 7.01–7.04 (m, 1H), 7.13–7.17 (m, 1H), 7.44 (d.d, 1H, *J* = 7.8 and 1.2Hz), 7.48–7.50 (m, 3H), 7.93 (d.d, 2H, *J* = 7.2 and 2.2 Hz); ^13^C NMR (100.49, CDCl_3_, 25°C) δ = 63.04, 115.67, 122.50, 126.57, 127.95, 128.78, 128.87, 128.90, 129.59, 131.29, 133.92, 135.60, 146.48, 158.84; IR (KBr): ν (cm^−1^) = 3055, 2849, 1612, 1480, 1274, 1216, 885, 753; MS (EI^+^): *m/z* = 209(100) [M^+^], 103 (98), 77 (89).

*3-(p-Methoxy)phenyl-1,4-benzoxazine* ***2*** *(BOX 2)*: ^1^H NMR (400, CDCl_3_, 25°C) δ = 3.87 (s, 3H), 5.04 (s, 2H), 6.91 (d, 1H, *J* = 8.0 Hz), 6.98 (d, 2H, *J* = 8.8 Hz), 7.01 (t, 1H, *J* = 8.0 Hz), 7.12 (t, 1H, *J* = 8.0 Hz), 7.40 (d, 2H, *J* = 8.0 Hz), 7.90 (d, 2H, *J* = 8.8 Hz); ^13^C NMR (100.49, CDCl_3_, 25°C) δ = 55.56, 62.86, 114.27, 115.59, 122.44, 127.61, 128.22, 128.30, 128.33, 134.11, 146.40, 158.25, 162.20; IR (KBr): ν (cm^−1^) = 3061, 2833, 1607, 1482, 1257, 1219, 831, 752; MS (EI^+^): *m/z* = 239(100) [M^+^], 224 (50), 133 (98), 77 (75).

*3-Phenyl-4′-methoxy-1,4-benzoxazine* ***3*** *(BOX 3)*: ^1^H NMR (400, CDCl_3_, 25°C) δ = 3.82 (s, 3H), 5.02 (s, 2H), 6.72–6.75 (m, 1H), 6.85 (d, 1H, *J* = 9 Hz) 7.03 (d, 1H, *J* = 2.7 Hz), 7.48–7.50 (m, 3H), 7.91–7.94 (m, 2H); ^13^C NMR (100.49, CDCl_3_, 25°C) δ = 55.98, 63.18, 112.06, 115.02, 116.02, 119.57, 126.67, 128.54, 128.92, 129.60, 131.35, 134.45, 140.45, 155.04, 159.68; IR (KBr): ν (cm^−1^) = 3064, 2834, 1597, 1491, 1266, 1208, 803, 755; MS (EI^+^): *m/z* = 239(100) [M^+^], 224 (49), 133 (98), 77 (75).

*3-(4-Benzo[b]* [1], [4] *oxazin-3-yl)phenyl)-benzo[b]* [1], [4] *oxazine* ***4*** *(BOX 4)*: ^1^H NMR (400, CDCl_3_, 25°C) δ = 5.11 (s, 4H), 6.93 (d, 2H, *J* = 8 Hz), 7.05 (t, 2H, *J* = 7.2 Hz), 7.18 (t, 2H, *J* = 7.6 Hz), 7.46 (d, 1H, *J* = 7.6 Hz), 8.03 (s, 4H); ^13^C NMR (100.49, CDCl_3_, 25°C) δ = 62.92, 115.78, 122.65, 126.93, 128.17, 129.25, 133.93, 137.68, 146.46, 157.72; IR (KBr): ν (cm^−1^) = 3060, 2919, 1609, 1479, 1280, 1208, 837, 745; MS (EI^+^): *m/z* = 340(100) [M^+^], 234 (78), 128 (50), 115 (66).

*3-(p-Methoxy)phenyl-4′-tert-butyl-1,4-benzoxazine* ***5*** *(BOX 5):* ^1^H NMR (400, CDCl_3_, 25°C) δ = 1.33 (s, 9H), 3.87 (s, 3H), 5.08 (s, 2H), 6.84 (d, 1H, *J* = 8.4 Hz), 6.98 (d, 2H, *J* = 8.8 Hz), 7.16 (d.d, 1H, *J* = 8.4 Hz, *J* = 2 Hz), 7.45 (d, 1H, *J* = 2 Hz), 7.89 (d, 2H, *J* = 8.8 Hz); ^13^C NMR (100.49, CDCl_3_, 25°C) δ = 31.62, 31.93, 55.56, 62.95, 114.26, 114.86, 124.66, 125.15, 128.28, 128.33, 129.40, 131.42, 144.00, 145.51, 158.31, 162.13; IR (KBr): ν (cm^−1^) = 2960, 1609, 1561, 1494, 1260, 834; MS (EI^+^): *m/z* = 295(87) [M^+^], 280 (93), 133 (100).

### Spectral profile and photostability

**Figure 3:**
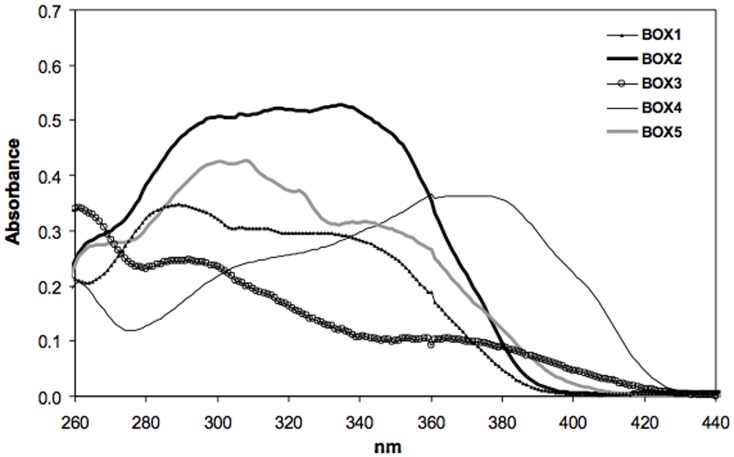
UV absorption spectra of 25 µM ethyl acetate solutions of the compounds tested.

The spectral profiles of 25 µM ethyl acetate solutions of BOX 1–5 are reported in Fig. 3. BOX 1 has no substituents on the benzoxazine ring nor on the phenyl group in position 3 and it absorbs throughout the whole UVB range and partly in the UVA one. However, by adding a methoxy group in the *para* position of the phenyl group (BOX 2), there is a remarkable increase in absorbance throughout the whole UVB/UVA range. When the same substituent is added in position 4 of the benzoxazine ring, the absorbance spectrum totally changes with respect to BOX 1: hardly no absorbance in the UVA region and only a slight one in the UVB range (BOX 3). By doubling the benzoxazine chromophore as in the case of BOX 4, there is a significant shift in absorbance to cover completely the UVA1 region (340–400 nm) whereas the addition of two substituents, i.e. a tert-butyl group on the benzoxazine ring and a methoxy group on the phenyl ring (BOX 5) does not lead to a substantial difference compared to BOX 1. There is only a very slight increment in absorbance in both the UVA and UVB ranges.

**Figure 4:**
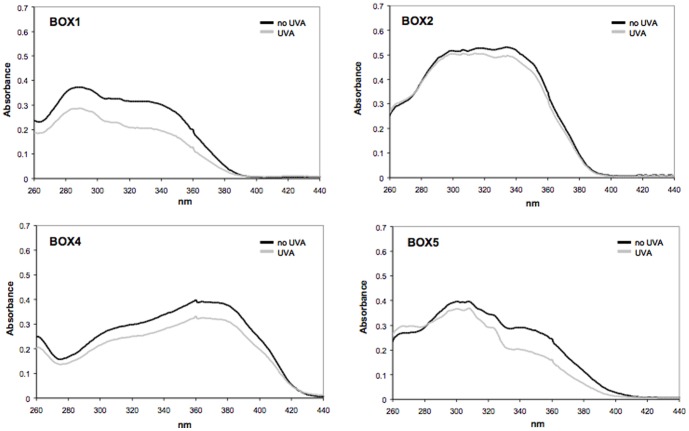
UV absorption spectra of compounds tested (25 µM) before (black line) and after (grey line) UVA exposure (275 kJ/m^2^), followed by extraction from liposomes with ethyl acetate. See materials and methods for experimental details.

The influence of UVA exposure on the spectral stability of the UV-filters incorporated in liposomes is shown in Fig. 4. BOX 3 was not examined since it's spectral profile was not deemed appropriate for consideration as a UV filter. The data show that BOX 2 and BOX 4 are totally photostable since there is no appreciable decrease in their absorbance spectra after exposure to 275 kJ/m^2^ UVA. BOX 1 and BOX 5 are less photostable as there is ∼35% loss in absorbance throughout the whole UVB/A range for BOX 1 and ∼27% absorbance decrease in the UVA range for BOX 5.

**Figure 5:**
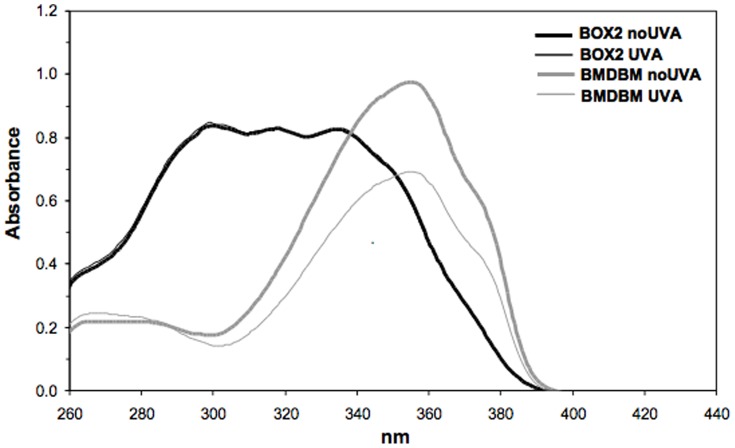
UV absorption spectra of formulations containing 6% concentrations of BOX2 or BMDBM, before (thick lines) and after (thin lines) UVA exposure (275 kJ/m^2^), followed by extraction with ethyl acetate. See materials and methods for experimental details.

Because of the high absorbance and spectral profile spanning both the UVB/UVA range of BOX 2, only this UV filter was chosen for subsequent photoprotection studies to ascertain protection at the molecular and cellular level of human dermal fibroblasts exposed to UVA. BOX 2 was incorporated into an oil/water formulation (6% w/w) since UV-filters should ultimately be tested under typical conditions of usage, i.e. formulated at a concentration ranging between 3–10% w/w. For comparative purposes, BMDBM was also formulated at the same concentration. The photostability of the two formulated UV-filters was first tested and Fig. 5 documents their UV absorbance spectra before and after UVA exposure. There is no decrease in spectral absorbance of BOX 2, confirming the photostability results reported in Fig. 4, whereas a substantial decrease in absorbance of BMDBM is observed. This loss in photostability is in accordance with previous reports on this UV filter present in certain sunscreen formulations [35], [36], and is due to the well known keto-enol isomerisation upon UV exposure which is followed by photo-cleavage and generation of free radicals [9], [37], [38].

### Cell viability assay

**Figure 6:**
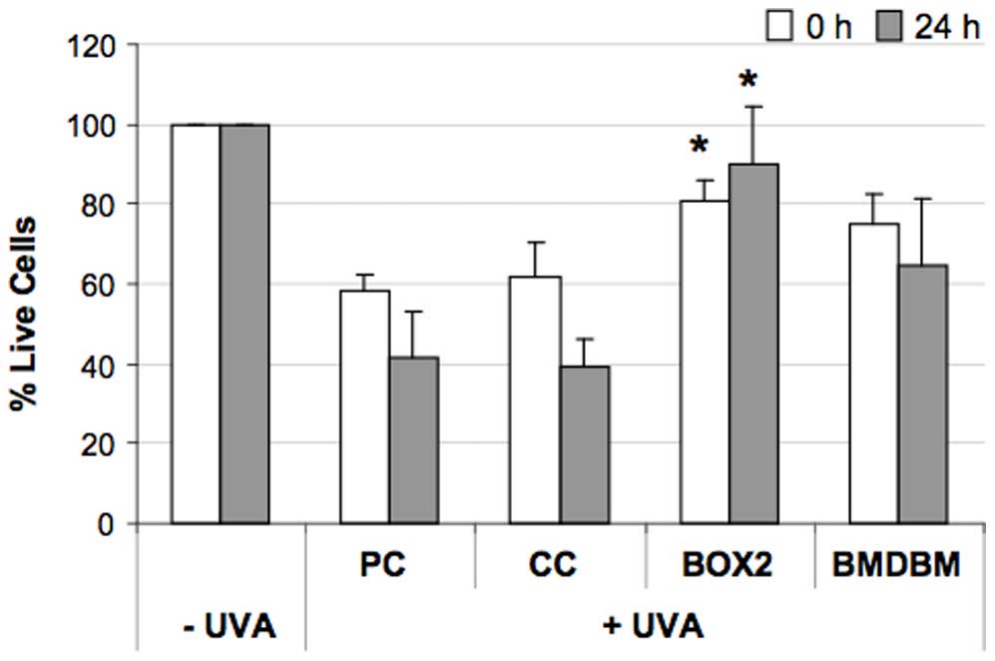
Viability of HDF exposed or not exposed to UVA (365 kJ/m^2^), determined using the MTT assay, at time 0 and after 24 h. HDF were either screened with formulations (BOX2, BMDBM), control cream (CC) or not screened at all (PC, positive control). Data are expressed as percentage of live cells compared to unexposed controls (−UVA). Error bars represent ± S.D. * vs PC.

The results on cell viability assessed using the MTT assay both at time 0, i.e. right after exposure and 24 h later, are reported in Fig. 6. Cell viability in the positive control (PC) decreases by about 50% both at 0 h and at 24 h, as can be expected. The cells screened by the control cream (CC) showed a similar behaviour, whereas those screened by the formulation containing BOX2 showed a significantly higher viability (∼80–90%) with respect to PC, both at 0 h and at 24 h. Instead, for the formulation prepared with BMDBM, although viability increases compared to PC, this is not significant. In addition, after 24 h the cell viability of samples screened with the cream containing BOX 2 appears to increase compared to 0 h indicating that the majority of cells are able to recover from UV damage, whereas for those screened with the cream containing BMDBM, cell viability appears to decrease after 24 h, suggesting that cells are more damaged and not able to recover as efficiently.

### Intracellular ROS assay

**Figure 7:**
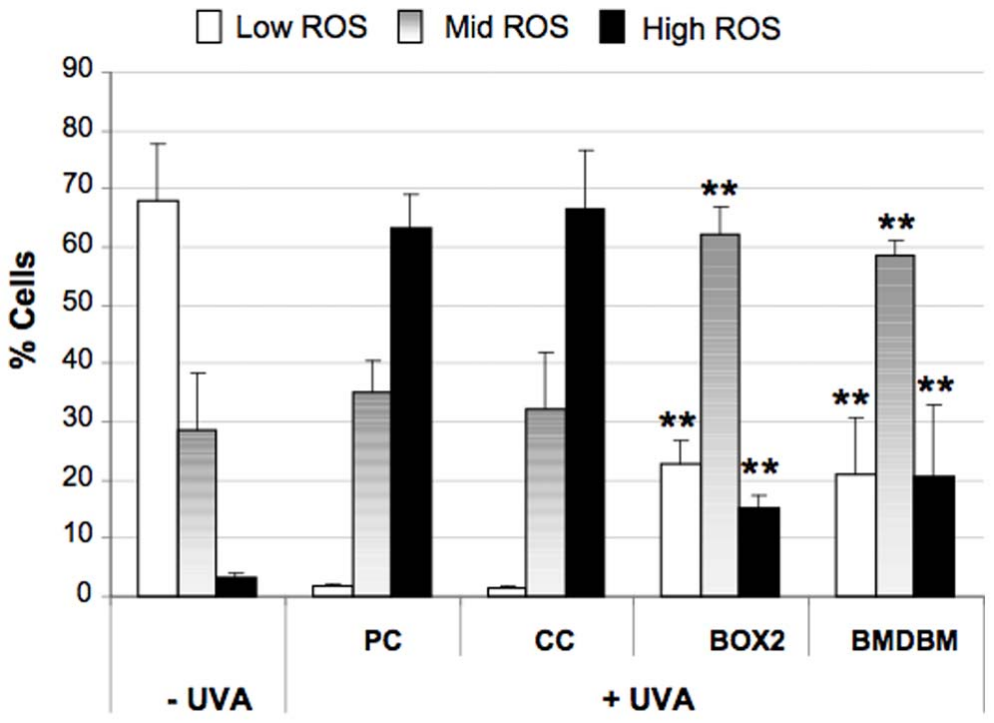
Flow cytometric analysis of intracellular levels of ROS in HDF, exposed or not exposed to UVA (365 kJ/m^2^), determined using the carboxy-DCFH-DA assay. HDF were either screened with formulations (BOX2, BMDBM), control cream (CC) or not screened at all (PC, positive control). Data are reported as percentage of cells presenting low (□), mid (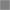) and high (▪) intracellular levels of ROS expressed in terms of carboxy-DCF fluorescence. Error bars represent ± S.D. ** vs PC.

Since UVA is known to produce ROS, the levels of these were measured intracellularly using the ROS-index probe carboxy-DCFH-DA. In order to better quantify the differences in intracellular ROS content, markers relative to low, mid and high green fluorescence channels were arbitrarily set and the percentage of cells belonging to each region was calculated. As shown in Fig. 7, after UVA exposure there is a considerable increase in intracellular ROS production in PC samples as well as in those screened with CC compared to the non-irradiated samples. In fact a low percentage of cells with low levels of ROS were detected, along with a concomitant high percentage of cells (∼65%) with high levels of ROS. The use of the formulations with BOX 2 or with BMDBM lead to a significant shift towards lower fluorescence values which lie in the region of mid ROS, implying reduced ROS production compared to the two former cases.

### Mitochondrial membrane potential assay

**Figure 8:**
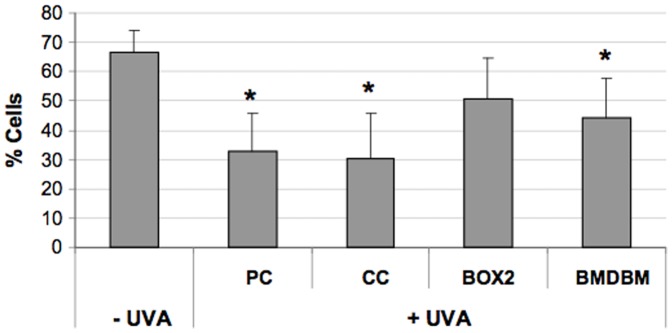
Flow cytometric analysis of mitochondrial membrane potential in HDF, exposed or not exposed to UVA (365 kJ/m^2^), determined using the JC-1 assay. HDF were either screened with formulations (BOX2, BMDBM), control cream (CC) or not screened at all (PC, positive control). Data are reported as percentage of cells presenting high mitochondrial membrane potential in terms of JC-1 red fluorescence. Error bars represent ± S.D. * vs −UVA.

Mitochondrial membrane potential was measured in terms of JC-1 fluorescence. The extent of depolarization was derived by evaluating the percentage of cells showing a decrease in red fluorescence. The data reported in Fig. 8 shows that after UVA exposure, there is a significant drop in mitochondrial membrane potential in PC samples and in those screened with CC compared to the non-irradiated negative control (-UVA). This decrease is less remarkable in samples screened with the cream containing BMDBM although still significant. Instead, membrane potential is highest in HDF screened with the BOX 2 cream, since membrane potential is restored to levels which are not significantly different from the non-irradiated control.

### Comet assay

**Figure 9:**
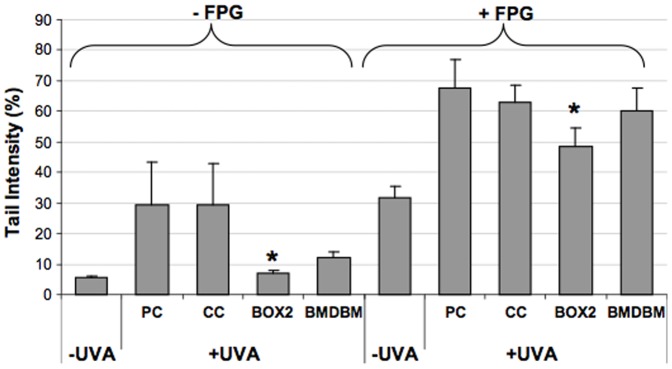
Cellular DNA damage in HDF, exposed or not exposed to UVA (365 kJ/m^2^), assessed using the standard alkaline comet assay (−FPG) and the modified version with FPG (+FPG) for detection of oxidised purines. HDF were either screened with formulations (BOX2, BMDBM), control cream (CC) or not screened at all (PC, positive control). Data are reported as the average of the median values of tail intensity. Error bars represent ± S.D. * vs PC.

This electrophoretic assay on single cells followed by staining with ethidium bromide was employed to assess DNA integrity and oxidation. With this assay, DNA damage can be detected and quantified at the level of each single cell by measuring the displacement of genetic material between the cell nucleus (comet “head”) and the resulting comet “tail”. The damage can be amplified by using the enzyme FPG which generates a gap in the DNA molecule at the level of oxidized purines. As can be observed in Fig. 9, both in the classic version of this assay (-FPG) and in that using FPG (+FPG), UVA exposure leads to a 30% increase in DNA damage in those samples screened with nothing (PC) or with the control cream (expressed as % tail intensity, i.e. % fluorescence of the tail in relation to the total fluorescence of the comet). In the presence of creams containing BMDBM or BOX 2, DNA damage is reduced almost to the level of the non-irradiated control. In the case of BOX 2 cream this decrease is significant when compared to the positive control (PC). With FPG, a greater amount of DNA damage can be detected, as expected, but the trend in the results obtained is the same as that observed without FPG.

### Gene expression by RT-qPCR

**Figure 10:**
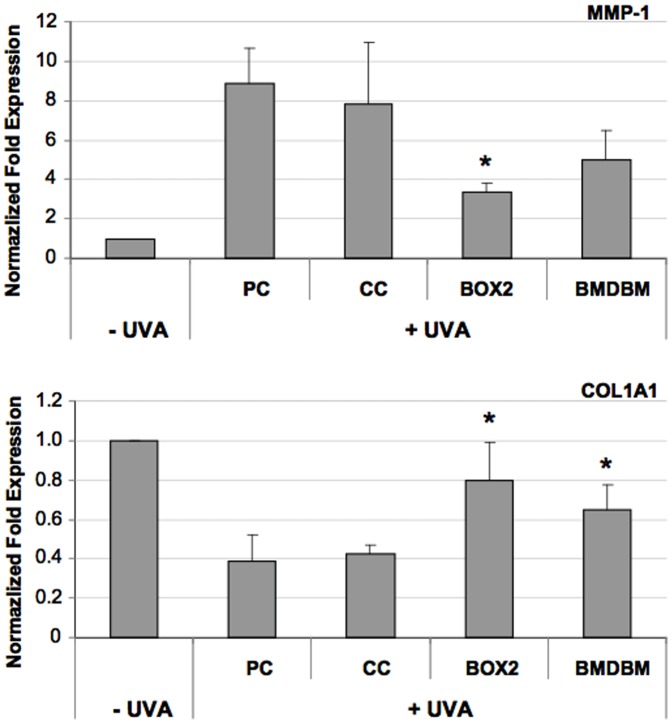
Gene expression analysis of MMP-1 and COL1A1 in HDF, exposed or not exposed to UVA (275 kJ/m^2^), assessed using qPCR. HDF were either screened with formulations (BOX2, BMDBM), control cream (CC) or not screened at all (PC, positive control). Data are reported as normalized fold expression using the 2^−ΔΔCt^ method. Error bars represent ± S.D. * vs PC.

UVA is also able to modulate gene expression, and at the level of the dermis where HDF reside, two important genes are modulated: MMP1 and COL1A1. COL1A1, the gene that expresses the synthesis for the α1 chain of type 1 collagen, the most abundant protein in skin connective tissue, is reduced, while the activity of MMP-1, which degrades it, is increased [39], [40]. In fact, Fig. 10 shows that following UVA exposure, the expression of MMP1 increases 8-fold in samples not screened by UV filters, but in those screened by BOX 2 and BMDBM creams, a decrease in gene expression can be observed. When compared with CC, this decrease results significant only in the samples screened by the cream formulated with BOX 2. In the case of COL1A1, expression of mRNA is reduced after UVA exposure, but when HDF are screened by the creams containing the UV filters, this reduction is less remarkable almost reaching the levels of the non-irradiated control. In both cases, the variation in gene expression is statistically significant compared to HDF screened by the control cream.

## Discussion

Photoprotection from repeated exposure to the sun's rays reaching the skin's surface is an essential preventive and therapeutic measure against photoageing and photocarcinogenesis. To this end, aside from wearing protective clothing, hats, glasses, and being sensible about sun exposure, the most popular method is through the use of topical sunscreens that contain UV filters as their active ingredients. Evaluation of the efficacy of sunscreens *in vivo* is based on their Sun Protection Factor (SPF) which is a measure of protection against sunburn or erythema, a biological response primarily due to UVA2 (320–340 nm) and UVB [27], [41]. Instead, *in vivo* UVA protection is based on the immediate (seconds) or persistent (2–24 h) pigmentation changes or on the minimal erythematous response and persistent pigmentation of the skin caused by UVA (IPD, PPD, UVA-PF methods, respectively) [26], [42], [43]. However, besides from being costly and time consuming, these *in vivo* methods raise ethical issues because of the potential damage to the skin of volunteers. Also, these parameters only reflect part of the UV-induced cascade of reactions in the skin. They give us no information about what actually happens at the cellular and molecular level of viable skin cells reached by UV rays that sunscreens do not manage to filter out. Furthermore, the SPF method has become under close scrutiny because of the anti-inflammatory effect of several UV filters present in current sunscreen products that can suppress erythema and hence yield a false impression of the amount of UV protection actually provided by the sunscreens containing them [44]. For the above motivations, *in vitro* testing is strongly recommended, as also exemplified by the recent ban on animal testing in Europe for cosmetic products and ingredients due to ethical reasons [45]. To this end, several *in vitro* techniques have already been developed, however at present there is no broadly accepted method [46].

The present study demonstrates that by using simple, *in vitro* tests such as those proposed here, the efficacy of UV filters and sunscreens at the cellular and molecular level, under conditions which mimic their actual application and exposure, can be investigated. In fact, to be efficient, a sunscreen should screen out as much as possible the UV rays reaching the skin, the majority of which are UVA (>95%). UVA rays are capable of penetrating into the dermis where fibroblasts reside and to induce cell damage via generation of ROS, through the photoexcitation of endogenous photosensitizers [2], [4]. UVA-induced oxidative damage mediated by increased ROS formation has consequences both at the molecular and cellular level. Cell viability can be compromised, mitochondrial function can be impaired, genotoxicity may arise through the formation of double- and single-strand breaks and oxidized purines, and the expression of important genes involved in the extracellular matrix of skin may be modulated [3], [47]. All these parameters can be tested in cultures of HDF after their exposure to UVA. If UVA rays are screened out by a sunscreen film placed on top of the cells, but not in contact with them, as in the experimental model used here, then these parameters will be positively affected. Indeed, the results obtained in our study demonstrate that the formulations containing the two UV filters tested individually, are able to filter out the UVA rays thus affecting the cellular and molecular endpoints considered. In fact, the results obtained when the formulation containing no UV filter was used, were similar to the positive control, i.e. no formulation. This model may mimic what should actually happen in practice when sunscreen is spread on skin. Sunscreens should not penetrate the skin and be systemically absorbed, but should be present as a uniform layer on top of the skin in order to consistently screen out harmful UV rays from penetrating within. Furthermore, not only the screening efficiency is tested, but simultaneously, after UVA exposure, the possible photodegradation of the UV filters present in the formulations prepared can be evaluated. Through solvent extraction and UV absorbance measurements, any changes before and after UVA irradiation can be easily observed and correlated with the endpoints investigated. The results reported in this study show that out of all the five possible UV filters synthesized, only two resulted completely photostable (BOX 2 and BOX 4) when incorporated in liposomes. The photostability of BOX 2 was then tested further by formulating it and comparing it with formulated BMDBM. The photostability of BOX 2 observed in liposomes was confirmed in the formulation. The greater photostability of BOX 2 and the broader UV absorbance spectrum compared to BMDBM, is the most likely explanation for the higher protective effects observed when the former UV filter was used in the formulation to protect the underlying HDF against UVA-induced damage. This is in line with the work of Lejeune et al. [48] who demonstrated by using human reconstructed skin *in vitro*, that efficient daily protection requires a high UVA filtration level which cannot be compensated by a high SPF value of a sunscreen. There are only a few other reports in the literature where sunscreen solutions or sunscreens have been applied on quartz slides/petri dishes or in between two quartz slides and placed over cell microplates of the same dimensions and irradiated [49]–[51]. Almost all of them have mainly focused on the expression of matrix metalloproteinases, particularly MMP-1, and only some have looked at UVA-induced DNA damage to evaluate sunscreen efficacy. In the present report not only expression of MMP-1 has been considered, but also that of COL1A1, which is just as determinant in skin photoageing as metalloproteinases. In addition, other biologically relevant endpoints related to the damaging effects of UVA on human skin fibroblasts were investigated (DNA integrity, intracellular ROS production, cell viability, mitochondrial function). Although all the information from an *in vitro* or from an *in vivo* method cannot be obtained for sunscreen global protection, the methods here employed do however give the essential information and the real possibility to test, compare and classify suncare products and individual UV filters, without being affected by anti-inflammatory ingredients present in current sunscreen products. Their photostability and ability to reduce the quantity of UVA rays reaching dermal skin cells can be determined, which is reflected in the protection of relevant biological parameters. Because the general trend in Europe and probably elsewhere in the future, is to replace human testing with *in vitro* approaches, then more studies are needed to validate and standardize methods for measuring *in vitro* sun protection. It is by bearing this in mind, that the present study was carried out with the aim to obtain as much meaningful information as possible on the efficacy of suncare products by using simple *in vitro* techniques. Lastly, the new UV filters synthesized could be considered for future applications in the field of UV protection, which is not only confined to the cosmetic field, but also to other fields, such as polymers, plastics, paints and textiles.
